# Assessing attitudes, access, barriers, and facilitators to multidisciplinary care in pediatric inflammatory bowel disease

**DOI:** 10.1002/jpr3.70120

**Published:** 2025-11-23

**Authors:** Nicole Davidson, Jennie G. David, Jennifer L. Dotson, Brendan Boyle, Ross M. Maltz, Hilary K. Michel, ImproveCareNow Pediatric IBD Learning Health System

**Affiliations:** ^1^ Nationwide Children's Hospital Columbus Ohio USA; ^2^ Department of Pediatrics, Section of Gastroenterology University of Arkansas for Medical Sciences and Arkansas Children's Hospital Little Rock Arkansas USA; ^3^ The Ohio State University College of Medicine Columbus Ohio USA

**Keywords:** Crohn's disease, ImproveCareNow, pharmacist, psychologist, ulcerative colitis

## Abstract

**Objective:**

Multidisciplinary care is recommended for pediatric inflammatory bowel disease (IBD). This study aims to describe provider attitudes, barriers, and facilitators regarding multidisciplinary care in pediatric IBD, and explore associations between multidisciplinary care access and center‐level factors.

**Methods:**

This is a cross‐sectional survey of pediatric gastrointestinal (GI) providers at centers registered with the ImproveCareNow (ICN) Learning Health System in the United States. Participants completed the survey via REDCap. Respondents provided demographic information and answered questions regarding their center's access and approach to multidisciplinary IBD care. Data were analyzed using descriptive statistics, chi‐squared, and Fisher's exact tests.

**Results:**

Sixty‐nine providers across 55 ICN centers were included. All participants felt multidisciplinary care was beneficial and 80% felt patients/families desired this care. Participants endorsed barriers including lack of support from institutional leadership (39%), limited access to providers (39%), and inadequate numbers of providers (35%). The most common team members were nurses (94%), dietitians (92%), social workers (67%), and nurse practitioners/physician assistants (67%). Multidisciplinary teams commonly provided care via telehealth (94%), standardized educational materials (84%), multidisciplinary visits (69%), new diagnosis education (65%), and transition programs (61%). Access to nurse practitioners/physician assistants, quality improvement specialists, multidisciplinary visits, and support groups were associated with increasing center size (*p* < 0.05).

**Conclusion:**

Pediatric GI providers have positive attitudes regarding IBD multidisciplinary care and perceive this care as important and desired. Access and barriers are variable among US ICN centers. Future work should seek to further understand and address barriers and evaluate the benefits of multidisciplinary care in pediatric IBD.

## Abstract (highlights)

**What is Known:**

- Multidisciplinary care is recommended for pediatric patients with inflammatory bowel disease (IBD).
- Access to multidisciplinary care may vary between centers due to a variety of barriers.

**What is New:**

- Pediatric gastrointestinal providers have positive attitudes toward IBD multidisciplinary care.
- While most multidisciplinary care teams included nurses, dietitians, social workers, and advanced practice providers, clinical pharmacists and case managers were frequently lacking.
- The most common barriers to multidisciplinary care in this survey included lack of institutional support and limited access to multidisciplinary providers.

## 1 INTRODUCTION

Inflammatory bowel disease (IBD) is a chronic immune‐mediated condition that can impact the psychosocial health and quality of life of patients, in addition to their physical health.^1^, ^2^ The incidence of IBD is increasing worldwide, with up to 25% of cases presenting under the age of 18.^3^, ^4^ Multidisciplinary care is recommended in the care of children and adolescents with IBD.^2^, ^5^, ^6^ Multidisciplinary care models for pediatric IBD have been described in the literature although provider attitudes and perceived barriers and facilitators to multidisciplinary care remain largely unknown.^5^, ^7^

To ensure equitable, multidisciplinary care for all pediatric patients with IBD, we must understand multidisciplinary care accessibility at the current moment, as well as perceived barriers preventing providers from delivering these services. Identification of barriers provides targets for interventions and may inform efforts to expand access to care, allowing more patients to achieve optimal medical and psychosocial outcomes. This study aims to describe provider attitudes, barriers, and facilitators regarding multidisciplinary care in pediatric IBD, and explore associations between access to multidisciplinary care and center‐level factors including number of patients with IBD.

## 2 METHODS

### 2.1 Ethics statement

This study was approved by the Institutional Review Board at Nationwide Children's Hospital.

### 2.2 Recruitment

This is a cross‐sectional survey of pediatric gastrointestinal (GI) and surgery providers (physicians and advanced practice providers) who practice at centers participating in the ImproveCareNow (ICN) Learning Health System in the United States (US). Surveys were administered between March and August of 2024. At the time of survey, there were 101 participating ICN centers caring for 31,497 actively enrolled patients with IBD. Providers were recruited via emails to the ICN network listserv, postings on the ICN website, and direct emails to ICN center leads. Providers outside of these centers and nonmedical providers were excluded. Participants were electronically consented and completed the survey via REDCap. No incentive was offered to participants for survey completion.

### 2.3 Survey

The survey was designed by a multidisciplinary team including pediatric GI medical and psychosocial providers. The survey had 58 total questions (Supplemental Digital Content S1).

All participants provided demographic information including gender, race, job title, ICN center, practice setting (i.e., rural, urban, suburban), and number of years in practice. They also answered questions on Likert scales regarding attitudes toward multidisciplinary care (options included strongly agree, agree, neutral, disagree, and strongly disagree) and perceived barriers to providing multidisciplinary care at their center (options included not at all difficult, somewhat difficult, and very difficult).

Center leads (of which each ICN center has at least one) answered additional survey questions regarding multidisciplinary care at their center such as number of pediatric GI providers and IBD‐focused providers at their institution, members of multidisciplinary team, and resources available to patients (e.g., telehealth, educational materials, support groups). These objective questions were asked of center leads only to avoid duplicate/conflicting responses from centers.

### 2.4 Additional data

Data provided by the ICN network and collected by centers participating in the ICN network included the number of actively enrolled patients at the time of the survey and the contact information for ICN center leads.

### 2.5 Statistical analysis

Descriptive statistics were used to summarize data. ICN centers were categorized based on the number of actively enrolled patients (small: 0–199 patients, medium: 200–399 patients, large: 400+ patients) to determine if center size has an impact on attitudes toward and access to multidisciplinary care. One center was excluded from this portion of the analysis due to not submitting the number of enrolled patients to ICN. Fisher's Exact or chi‐square tests were performed using GraphPad Prism version 10 for Windows, GraphPad Software, www.graphpad.com to assess for associations between number of patients with IBD per center and access to team members and resources as well as attitudes regarding and barriers to multidisciplinary care.

## 3 RESULTS

### 3.1 Demographics

Eighty‐one providers responded to the survey, with 12 excluded due to not meeting inclusion criteria of being a medical provider. Of those who met inclusion criteria, there were 69 medical providers including 52 ICN center leads representing a total of 55 ICN centers (54% of US ICN centers at the time of this study).

Participating centers had an average of 15 GI physicians (standard deviation [SD] ± 11) per division. Roughly half (54%) of respondents considered their position to be primarily IBD‐focused (Table 1). Practice location was 71% urban, 27% suburban, and 2% rural. Practice setting was 75% academic, 17% large health system, 4% private practice, and 4% other (mixed practice model, multisubspeciality clinic) (Table 1). There was a statistically significant relationship between the number of ICN enrolled patients and total number of providers in the division (*p* = 0.002) and number of dedicated IBD providers (*p* ≤ 0.001).

**Table 1:** Demographics.

Centers surveyed (*n* = 101)	
Responses included	69
Number of centers included	55
Number of site leads	52
Practice location (*n* [%]); (*N* = 52)	
Urban	37 (71)
Suburban	14 (27)
Rural	1 (2)
Practice setting (*n* [%]); (*N* = 52)	
Academic	39 (75)
Large health system	9 (17)
Private	2 (4)
Other*	2 (4)
Respondent roles (*n*); (*n* = 69)	
GI attending	65
NP/APN	2
Surgeon	1
Retired attending	1
Respondent demographics	
# of years practicing (mean [SD])	16 (10)
Primarily IBD focused (*n* [%])	37 (54)
Female (*n* [%])	30 (43)
Non‐Hispanic (*n* [%])	63 (91)
# physicians in division (mean [SD])	15 (11)
% of physicians in division dedicated to IBD care (mean (SD))	15% (17%)

*Note*: *n* = sample size; other* = mixed practice model, multisubspecialty clinic.

Abbreviations: IBD, inflammatory bowel disease; NP/APN, nurse practitioner/advanced practice nurse; SD, standard deviation.

### 3.2 Perspectives on multidisciplinary care

**Figure 1:**
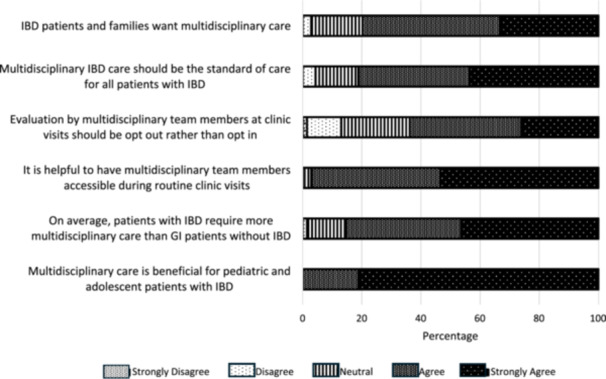
Attitudes toward multidisciplinary IBD care. GI, gastrointestinal; IBD, inflammatory bowel disease.

All survey participants felt that multidisciplinary care was beneficial for patients with IBD, with nearly all participants (97%) endorsing that it is helpful to have access to multidisciplinary providers during clinic. Eighty‐six percent of providers agreed with the statement that patients with IBD required more multidisciplinary care than other GI patients. Eighty‐one percent felt multidisciplinary care should be standard of care for patients with IBD and 80% felt that patients and families also desired this type of care. Sixty‐four percent of respondents felt that evaluation by multidisciplinary team members at clinic visits should be opt‐out rather than opt‐in (Figure 1).

### 3.3 Barriers to multidisciplinary care

**Figure 2:**
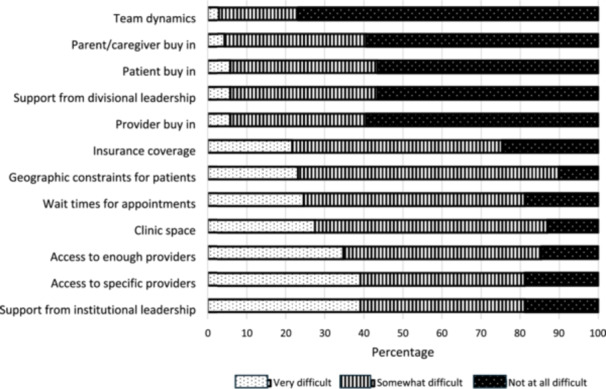
Perceived barriers to multidisciplinary IBD care. IBD, inflammatory bowel disease.

Participants endorsed various barriers to multidisciplinary care (defined as a response of “very difficult”), including lack of support from institutional leadership (39% of total participants; 50% of participants from small institutions, 41% medium, 33% large), limited access to multidisciplinary providers (39%; 50% small, 41% medium, 25% large), challenges securing adequate numbers of providers to meet clinical demand (35%; 50% small, 38% medium, 17% large), and/or inadequate clinic space (28%; 21% small, 31% medium, 25% large). There was no statistically significant difference identified in the most common barriers (defined as those with >25% response of “very difficult”) and ICN center size (all *p*‐values > 0.05). Less common barriers included wait times for appointments (25%), geographic constraints for patients (23%), inadequate insurance coverage (22%), lack of provider buy‐in (6%), lack of support of divisional leadership (6%), lack of patient or parent/caregiver buy‐in (6% and 4%, respectively), and team dynamics (3%) (Figure 2).

### 3.4 Multidisciplinary team members and processes

The most common multidisciplinary IBD team members reported by center leads included nurses (involved at 94% of centers), dietitians (92%), social workers (67%), nurse practitioners/physician assistants (67%), psychologists (61%), research coordinators (61%), prior authorization specialists/biologic navigators (51%), and IBD surgeons (51%). Less than half of centers reported availability of a data/quality improvement (QI) specialist (47%), clinical pharmacist (29%), and case manager (8%). Increasing center size was significantly associated with improved access to nurse practitioners/physician assistants, with significant differences in access between both small and medium‐sized centers and between small and large centers (*p* < 0.05). Access to data/QI specialists was also significantly associated with center size, driven by a significant differences between small and large centers (*p* < 0.05) (Table 2).

**Table 2:** Relationship between number of active IBD patients and aspects of multidisciplinary care.

Variable	Total (*n* = 51); *n* (%)	0–199 patients (*n* = 11); *n* (%)	200–399 patients (*n* = 22); *n* (%)	400+ patients (*n* = 18); *n* (%)	*p* value
Multidisciplinary team members					
Nurse	48 (94)	10 (91)	21 (95)	17 (94)	>0.99
Dietician	47 (92)	10 (91)	21 (95)	16 (89)	0.82
Social worker	34 (67)	7 (64)	16 (73)	11 (61)	0.74
Nurse practitioner/physician assistant	34 (67)	3 (27)	17 (77)	14 (78)	**0.02**
Psychologist	31 (61)	4 (36)	15 (68)	12 (67)	0.20
Research coordinator	31 (61)	6 (55)	12 (55)	13 (72)	0.47
PA/biologic navigator	26 (51)	3 (27)	11 (50)	12 (67)	0.12
IBD surgeon	26 (51)	4 (36)	11 (50)	11 (61)	0.48
Data/QI specialist	24 (47)	1 (9)	10 (45)	13 (72)	**<0.01**
Clinical pharmacist	15 (29)	1 (9)	7 (32)	7 (39)	0.20
Case manager	4 (8)	0 (0)	3 (14)	1 (6)	0.53
Aspects of multidisciplinary care at center					
Telehealth	48 (94)	10 (91)	20 (91)	18 (100)	0.43
Standardized education materials	43 (84)	8 (73)	19 (86)	16 (89)	0.54
MultiD visits	35 (69)	3 (27)	16 (73)	16 (89)	**<0.01**
Standardized education process for new diagnoses	33 (65)	5 (45)	16 (73)	12 (67)	0.29
Standardized transition program	31 (61)	7 (64)	10 (45)	14 (78)	0.11
Parent/caregiver support groups	20 (39)	1 (9)	7 (32)	12 (67)	**0.01**
Patient support groups	29 (37)	2 (18)	6 (27)	11 (61)	**0.03**

*Note*: Bold values indicate statistically significant results *p* < 0.05.

Abbreviations: IBD, inflammatory bowel disease; multiD, multidisciplinary; PA, prior authorization; QI, quality improvement.

Nearly all center leads reported that their teams provided IBD care via telehealth (94%), with involved providers including physicians (*N* = 47, 98%), dietitians (*N* = 34, 71%), nurse practitioners/physician assistants (*N* = 30, 63%), psychologists (*N* = 17, 35%), nurses (*N* = 11, 23%), and social workers (*N* = 10, 13%). Telehealth was most commonly used for teaching/educational visits (*N* = 24, 51%) and less commonly for multidisciplinary visits (*N* = 7, 15%). Sixty‐nine percent of centers reported conducting multidisciplinary visits, most often for routine office visits (74% of centers), health maintenance visits (37%), and teaching/education visits (31%). Other services offered by centers included the use of standardized educational materials (84%), new diagnosis educational processes (65%), and a transition program to adult care (61%); less than half of centers offered IBD support groups for parents/caregivers (43%) or patients (37%). The use of multidisciplinary visits was significantly associated with increasing center size, with significant differences in access between both small and medium‐sized centers and between small and large centers (*p* < 0.05). Access to both parent/caregiver and patient support groups were also associated with center size, driven by differences between small and large centers (*p* < 0.05) (Table 2).

## 4 DISCUSSION

In a nationwide survey of IBD centers participating in the ICN Learning Health System, the majority of survey participants felt that multidisciplinary care should be standard of care for children with IBD, and that patients and families desire this type of care as well. However, healthcare providers endorsed a variety of barriers to delivering multidisciplinary care including inadequate number of and lack of access to providers and lack of support from institutional leadership. The makeup of the multidisciplinary team and resources available also varied between centers, raising concerns for inequitable care delivery across the country.

Nearly all participants in this survey agreed with the general recommendations that children with IBD are best managed by a collaborative, multidisciplinary team. These findings are similar to those in a recent survey study where 92% of pediatric healthcare providers reported that the provision of psychosocial care to youth with IBD was very important.^8^ This model has the potential to increase the comprehensiveness of care delivered, decrease fragmentation in care, and improve communication between providers, ultimately improving patient outcomes.

In addition, it is known that patients with IBD have higher healthcare costs compared to the general population, and pediatric patients with IBD have been shown to have higher costs than their adult counterparts.^9^ Providing multidisciplinary may decrease overall healthcare utilization and lead to cost savings in the long run, as has been demonstrated in the adult IBD medical home model.^7^, ^10^, ^11^ Participants also thought that patients and families desired multidisciplinary care. Existing literature surrounding patient satisfaction and desire for multidisciplinary care supports a similar conclusion.^12^, ^13^, ^14^ Further research, including patient and caregiver facing work, is needed to determine how to personalize and deliver multidisciplinary care most effectively.

Despite near universal agreement that multidisciplinary care is important in pediatric IBD and perceived to be desired by patients and families, a variety of barriers to accessing this care were reported, with no significant differences in barriers cited by center size. The most common barriers were related to lack of support from institutional leadership and difficulty accessing adequate providers to meet clinical demand. Eighty‐one percent of providers 81% cited that lack of institutional support made providing multidisciplinary care very or somewhat difficult. Previous work comments on the importance of leveraging institutional support to build a multidisciplinary IBD program, but also acknowledges funding as a significant limitation, in addition to the amount of time and effort that can be required to build these programs and relay their usefulness to financial decision makers at individual pediatric institutions.^15^, ^16^ Additional research that demonstrates the impact of multidisciplinary care on clinical outcomes and cost effectiveness in the pediatric setting may bolster support from institutional leadership and increase access.

Though not one of the most frequently reported barriers to provision of multidisciplinary care, adequate insurance coverage for services was reported by nearly a quarter of participants. As the cost of managing IBD continues to grow, it is important that we ensure services are covered adequately to ensure our patients and their families are not burdened by additional fees. The 2024 position paper from the American Gastroenterological Association on the future of IBD care also cites insurance coverage of multidisciplinary services as a barrier to optimizing care, along with the inability for some providers to bill for their services, and thus difficulty financially supporting their position.^17^ Previously published work in pediatrics supports the same conclusion; insurance barriers, including lack of insurance, underinsurance, or inability of particular providers to accept specific insurance types has been cited as a barrier to receipt of multidisciplinary care, especially mental health and dietetic care.^18^, ^19^

The second most commonly cited barrier to providing multidisciplinary pediatric IBD care was related to limitations in the number and availability of multidisciplinary care team members, similar to data reported in a recent survey of pediatric GI healthcare providers focused on engagement with psychosocial care in pediatric IBD.^8^ Adequate access to pediatric dietitians for children with chronic GI diseases has also been reported in the literature.^18^ In our prior work on the pediatric IBD medical home model, we discuss ways in which centers might think creatively about roles within a multidisciplinary care model and leverage current team members to fulfill variable roles when other members are not available; for example, a social worker may be able to screen for anxiety and depression and refer a patient for behavioral health services if a psychologist is not available.^7^ Interestingly, in our study, providers did not find lack of provider, patient, or caregiver buy‐in as barriers to the provision of multidisciplinary care, which contrasts with the findings of the aforementioned survey study regarding attitudes toward psychosocial care in pediatric IBD, where these were frequently cited barriers.^8^

In our study, the make‐up of the multidisciplinary care team was variable by center. Nearly all centers had access to nurses and dietitians and the majority of centers had access to psychology and social work. A minority of centers had access to clinical pharmacists and QI/data specialists. Similar variability in multidisciplinary care has been reported in prior pediatric and adult studies.^5^, ^20^, ^21^ Our findings are encouraging in part; nurses can play an essential role in patient education, care coordination, and as a primary contact for patients and families.^22^, ^23^ While there is not an abundance of primary literature describing the role of a dietitian on the multidisciplinary IBD team, malnutrition and nutritional deficiencies are common pediatric IBD, and thus their abilities to conduct nutritional assessments, screen for and treat micronutrient deficiencies, promote growth and development, and provide guidance to those who choose to use diet as primary or adjunctive therapy are essential.^24^, ^25^, ^26^ The impact of psychosocial factors on the health of children with IBD is well known, and many prior studies have emphasized the importance of psychosocial care for patients with IBD.^27^, ^28^ Access to psychosocial providers allows for assessment and management of comorbid behavioral health disorders, coping with chronic illness, perioperative support, and troubleshooting medication adherence.^8^, ^29^

However, we should note that over one‐third of centers did not have access to a psychosocial provider exposing an important gap in care. Additionally, access to these providers does not indicate the amount of time providers are allotted for IBD care, as they may have responsibilities to other patient populations within GI and beyond. Very few centers had access to a clinical pharmacist. Recently, the expanding roles of the clinical pharmacist in IBD care has been described and includes medication counseling, assistance with prior authorization and appeals process, therapeutic drug monitoring, and biosimilar education.^30^, ^31^ As the armamentarium of IBD pharmaceuticals grows and the insurance landscape becomes more complex, this gap in access at the vast majority of surveyed US ICN centers is an area for growth. Finally, our study revealed variable access to certain providers by center size, with larger centers having increased access to dedicated IBD providers, nurse practitioners/physician assistants and data/QI specialists. This raises concern for differences in care at centers with smaller numbers of patients with IBD and should be an area of further study.

In addition to inquiring about team member access, our survey asked about mechanisms of care delivery. During the COVID‐19 pandemic, many centers converted to telehealth for use in pediatric IBD care, and have maintained this practice even after the return of availability of in person services to expand access.^32^, ^33^ Many of these changes made during the pandemic have persisted into the present, with 94% of our survey respondents reporting the use of telehealth at their center. While primarily used by physicians, advanced practice providers, and dietitians, a small portion of centers reported other multidisciplinary team members making use of telemedicine, primarily for education‐related visits. Future work should examine more granular details about how telemedicine is being used (i.e., entire multidisciplinary visits being conducted via telemedicine *vs.* using it to increase access to individual team members), as a potential way to navigate access‐related barriers. We also inquired about access to support groups, which were more commonly accessible at larger centers. Literature on pediatric IBD specific peer and parent mentoring groups is limited, but similar groups in the adult setting or with other disease processes have demonstrated positive impact.^34^, ^35^, ^36^ Once again, variable access at smaller centers raises the concern for inequity in care, though online and virtual peer support may improve this ^37^

It is important to note that there is not one standard to follow for provision of multidisciplinary IBD care, and models may differ center to center based on available team members and resources. Several centers have described their pediatric IBD programs in the literature recently, resources which could be used as starting points for those seeking to grow their programs.^5^, ^7^ Both publications highlight multidisciplinary visits delivered in person or via telemedicine to expand access. Purposes of such visits include education at the time of diagnosis, annual health maintenance visits, and delivery transition readiness programs. Some programs also send psychosocial screeners to patients and families before visits to help target visit content.^5^, ^7^ No matter what the format, clear communication of assessments and recommendations between providers, patient, and family is essential.

Limitations of this study include the subjective nature of some survey questions, and that while we inquired about multidisciplinary care, this survey did not delve into the level of collaboration between multidisciplinary team members, only their participation. Additionally, this survey was limited to ICN centers in the United States, with approximately half of the centers responding, limiting generalizability to non‐ICN centers and those outside of the United States.

## 5 CONCLUSION

Pediatric GI providers have positive attitudes regarding IBD multidisciplinary care and perceive this care as important and desired by their patients and caregivers. However, access to this care is variable and sometimes absent across US ICN centers, with centers with fewer than 200 patients appearing to have less access. Reported barriers exist, the most common of which are inadequate institutional support and access to providers. As the incidence and prevalence of pediatric IBD increase along with the complexity of care provided, growing and improving access to the multidisciplinary team is essential to optimize outcomes.^3^, ^38^ Future work should focus on examining benefits of multidisciplinary pediatric IBD care as well as associated cost savings, which may improve institutional support and improve accessibility across the United States.

## CONFLICT OF INTEREST STATEMENT

The authors declare no conflicts of interest.

## Supporting information

survey PDF.
