# Supplementary material for: Assessing attitudes, access, barriers, and facilitators to multidisciplinary care in pediatric inflammatory bowel disease
Source: JPGN Rep. 2025 Nov 23;7(1):90–8. doi: 10.1002/jpr3.70120 (PMC12894075; doi:10.1002/jpr3.70120)
Supplement: Supplementary file 1 — survey PDF. [file JPR3-7-90-s001.pdf]

At which ImproveCareNow center in the United States do you work?

- ◯ AL: Alabama Children's of Alabama
- ◯ AL: University of South Alabama
- ◯ AZ: Phoenix Children's Hospital
- ◯ AK: Arkansas Children's Hospital
- ◯ CA: CHOC Children's Hospital of Orange County
- ◯ CA: Children's Hospital Los Angeles
- ◯ CA: Cottage Children's Medical Center
- ◯ CA: MemorialCare Miller Children's and Women's Hospital
- ◯ CA: Rady Children's Hospital - San Diego
- ◯ CA: Stanford Children's Health
- ◯ CA: UC Davis Children's Hospital
- ◯ CA: UCSF Benioff Children's Hospital
- ◯ CA: Valley Children's Health Care
- ◯ CO: Children's Hospital Colorado
- ◯ CT: Yale-New Haven Children's Hospital
- ◯ DC: Children's National Health System
- ◯ DE: Nemours Children's Health System - Wilmington
- ◯ FL: Arnold Palmer Hospital for Children
- ◯ FL: Golisano Children's Hospital of Southwest Florida
- ◯ FL: Holtz Children's Hospital at University of Miami-Jackson Memorial Medical Center
- ◯ FL: Nemours Children's Specialty Care - Jacksonville
- ◯ FL: Nemours Children's Hospital - Orlando
- ◯ FL: Nicklaus Children's Hospital
- ◯ FL: UF Health Pediatric Gastroenterology, Hepatology & Nutrition
- ◯ GA: Children's Healthcare of Atlanta at Egleston/Emory University
- ◯ GA: Children's Healthcare of Atlanta at Scottish Rite- GI Care for Kids
- ◯ IA: University of Iowa Stead Family Children's Hospital
- ◯ IL: Advocate Children's Hospital, Park Ridge
- ◯ IL: Ann & Robert H. Lurie Children's Hospital of Chicago
- ◯ IL: Children's Hospital of Illinois - University of Illinois at Peoria
- ◯ IN: Riley Hospital for Children Kentucky
- ◯ KY: Kentucky Children's Hospital
- ◯ KY: University of Louisville
- ◯ LA: Children's Hospital New Orleans
- ◯ LA: Ochsner for Children
- ◯ ME: Barbara Bush Children's Hospital at Maine Medical Center
- ◯ MA: Boston Children's Hospital
- ◯ MA: MassGeneral Hospital for Children
- ◯ MI: Bronson Children's Hospital
- ◯ MI: Helen DeVos Children's Hospital
- ◯ MI: University of Michigan - C.S. Mott Children's Hospital
- ◯ MN: Mayo Clinic
- ◯ MN: University of Minnesota
- ◯ MO: Cardinal Glennon Children's Medical Center, St. Louis University
- ◯ MO: Children's Mercy
- ◯ MO: St. Louis Children's Hospital - Washington University
- ◯ NE: Children's Nebraska
- ◯ NH: Dartmouth Health Children's
- ◯ NH: New Hampshire Hospital for Children (NHHC)
- ◯ NJ: Joseph M. Sanzari Children's Hospital at Hackensack Meridian Health
- ◯ NY: Kravis Children's Hospital at Mount Sinai
- ◯ NY: NewYork-Presbyterian Komansky Children's Hospital / Weill Cornell Medical Center
- ◯ NY: NYU Langone Medical Center Hassenfeld

	Children's Hospital<br>◯ NY: NYU Pediatric Gastroenterology - Lake Success<br>◯ NY: University of Rochester, Golisano Children's Hospital<br>◯ NY: Upstate Golisano Children's Hospital<br>◯ NY: Stony Brook Children's Hospital<br>◯ NY: The Children's Hospital at Montefiore<br>◯ NV: Pediatric Gastroenterology & Nutrition Associates<br>◯ NC: Duke Children's Hospital and Health Center<br>◯ NC: Levine Children's Hospital<br>◯ NC: University of North Carolina at Chapel Hill<br>◯ OH: Akron Children's Hospital<br>◯ OH: Cincinnati Children's Hospital Medical Center<br>◯ OH: Cleveland Clinic Children's<br>◯ OH: Dayton Children's Hospital<br>◯ OH: Nationwide Children's Hospital<br>◯ OH: Rainbow Babies and Children's Hospital<br>◯ OK: Oklahoma University Medical Center<br>◯ OR: Randall Children's Hospital<br>◯ OR: OHSU Doernbecher Children's Hospital<br>◯ PA: Geisinger Janet Weis Children's Hospital<br>◯ PA: Penn State Hershey Children's Hospital<br>◯ PA: The Children's Hospital of Philadelphia<br>◯ PA: UPMC Children's Hospital of Pittsburgh<br>◯ SC: MUSC Children's Hospital<br>◯ SC: Prisma Health - Midlands<br>◯ SC: Prisma Health - Upstate<br>◯ TN: Le Bonheur Children's Hospital, Memphis, TN<br>◯ TN: Monroe Carell Jr. Children's Hospital at Vanderbilt<br>◯ TN: Children's Hospital at Erlanger<br>◯ TX: Children's Memorial Hermann Hospital - UT Houston<br>◯ TX: Cook Children's Medical Center<br>◯ TX: Dell Children's Medical Center of Central Texas<br>◯ TX: UT Southwestern / Children's Hospital<br>◯ VA: Bon Secours St. Mary's Children's Hospital<br>◯ VA: Carilion Children's<br>◯ VA: Children's Hospital of The King's Daughters<br>◯ VA: Children's Hospital of Richmond at VCU<br>◯ VA: Pediatric Specialists of Virginia<br>◯ VA: University of Virginia Children's Hospital<br>◯ VT: The University of Vermont Children's Hospital<br>◯ WA: Mary Bridge Children's Network<br>◯ WA: Providence Sacred Heart Children's Hospital<br>◯ WA: Seattle Children's Hospital<br>◯ WI: American Family Children's Hospital<br>◯ WI: Children's Hospital of Wisconsin<br>◯ WV: WVU Medicine Children's<br>◯ I don't belong to an ICN center<br>◯ I belong to an ICN center outside the US
Are you the ICN center lead/co-lead?	◯ Yes<br>◯ No
What is your current role?	◯ Pediatric gastroenterologist (attending physician)<br>◯ Pediatric gastroenterology fellow<br>◯ Nurse Practitioner (NP)/Advanced Practice Nurse (APN)<br>◯ Physician Assistant (PA)<br>◯ Other ______

How many years have you been practicing pediatric gastroenterology?

1 year — 50 years

(Place a mark on the scale above)

What percentage of your clinical time is spent caring for patients with IBD?

- ◯ < 10%
- ◯ 10-25%
- ◯ 26-50%
- ◯ 51-75%
- ◯ >75%
- ◯ Unknown

Do you consider your position to be primarily IBD focused?

- ◯ Yes
- ◯ No

Please identify your gender

- ◯ Female
- ◯ Male
- ◯ Transgender
- ◯ Non-binary
- ◯ Other ______
- ◯ Prefer not to say

Please identify your ethnicity

- ◯ Non-Hispanic
- ◯ Hispanic
- ◯ Prefer not to say

Please identify your race

- ◯ Caucasian/White
- ◯ Black or African American
- ◯ Asian
- ◯ American Indian
- ◯ Pacific Islander
- ◯ Multiracial
- ◯ Other ______
- ◯ Prefer not to say

The following questions are about your care center and how IBD care is delivered there. Multidisciplinary care refers to healthcare providers from different disciplines working together to optimize care for patients and their families.

How would you describe the main location of your primary hospital or practice?

- ◯ Urban
- ◯ Suburban
- ◯ Rural

What is the setting of your current hospital or practice?

- ◯ Academic
- ◯ Private practice
- ◯ Large health system practice (i.e. Kaiser)
- ◯ Military
- ◯ Military treatment facility
- ◯ Other ______

How many physicians are in your division?

____

How many physicians are dedicated primarily to IBD care?

____

Which of the following providers routinely participate in the care of patients with IBD at your center? Please indicate how many of each provider are at your center, if applicable.	☐ Nurse practitioner ______<br>☐ Physician assistant ______<br>☐ Clinical pharmacist ______<br>☐ Dietician ______<br>☐ Nurse ______<br>☐ Psychologist ______<br>☐ Social Worker ______<br>☐ IBD specialized surgeon ______<br>☐ Data or quality improvement Specialist ______<br>☐ Research coordinator ______<br>☐ Prior authorization specialist/biologic navigator ______<br>☐ Case manager ______<br>☐ Other ______
Does your center currently utilize telehealth for IBD related visits?	◯ Yes<br>◯ No
If yes, which providers utilize telehealth? Check all that apply	☐ Physician<br>☐ Nurse practitioner<br>☐ Physician assistant<br>☐ Clinical pharmacist<br>☐ Dietician<br>☐ Nurse<br>☐ Psychologist<br>☐ Social Worker<br>☐ IBD specialized surgeon<br>☐ Data or quality improvement Specialist<br>☐ Research coordinator<br>☐ Prior authorization specialist/biologic navigator<br>☐ Case manager<br>☐ Other ______
If yes, for which types of visits is telehealth used? Check all that apply.	☐ Routine follow up visits<br>☐ Multidisciplinary visits<br>☐ Teaching/educational visits<br>☐ Other ______
Does your center have a standardized educational process for patients newly diagnosed with IBD?	◯ Yes<br>◯ No
If yes, please describe the context in which you provide education.	☐ Individual education (single patient/family)<br>☐ Group education (multiple patients/families)<br>☐ Other ______
If yes, which team members are involved? Check all that apply	☐ Nurse practitioner<br>☐ Physician assistant<br>☐ Clinical pharmacist<br>☐ Dietician<br>☐ Nurse<br>☐ Psychologist<br>☐ Social Worker<br>☐ IBD specialized surgeon<br>☐ Physician<br>☐ Case Manager<br>☐ Other ______
Do you provide standardized IBD education materials to patients/families?	◯ Yes<br>◯ No

If yes, which of the following educational material types do you routinely use? Check all that apply	☐ Center specific resources<br>☐ Widely available resources ______
If yes, when do you provide these materials? Check all that apply	☐ At diagnosis<br>☐ Prior to medication initiation/medication change<br>☐ During preparation for surgery<br>☐ In preparation for transfer/transition of care<br>☐ Other ______
Does your center offer multidisciplinary IBD visits? "Multidisciplinary" in this case refers to care visits with multiple providers available to see the patient at the same time or sequentially.	◯ Yes<br>◯ No
If yes, for which type of visits?	☐ Routine office visits<br>☐ Teaching/educational visits<br>☐ Health maintenance visits<br>☐ IBD-related surgical visits<br>☐ Other ______
If yes, which team members are involved? Check all that apply	☐ Nurse practitioner<br>☐ Physician assistant<br>☐ Clinical pharmacist<br>☐ Dietician<br>☐ Nurse<br>☐ Psychologist<br>☐ Social Worker<br>☐ IBD specialized surgeon<br>☐ Physician<br>☐ Case Manager<br>☐ Other ______
Does your center have a standardized transition program for adolescents and young adults with IBD preparing to transfer to adult care?	◯ Yes<br>◯ No
Is the transition program multidisciplinary?	◯ Yes<br>◯ No
If yes, which providers are involved?	☐ Nurse practitioner<br>☐ Physician assistant<br>☐ Clinical pharmacist<br>☐ Dietician<br>☐ Nurse<br>☐ Psychologist<br>☐ Social Worker<br>☐ IBD specialized surgeon<br>☐ Physician<br>☐ Case Manager<br>☐ Other
Does your center have IBD parent/caregiver support groups or programs?	◯ Yes<br>◯ No
If yes, please describe	________________________________

Does your center have IBD patient support groups or programs?

◯ Yes
◯ No

If yes, please describe

____

These questions seek to understand attitudes, barriers, and facilitators to providing multidisciplinary care to children and adolescents with IBD. Multidisciplinary care refers to healthcare providers from different disciplines working together to optimize care for patients and their families.

When it comes to multidisciplinary care for children and adolescents with IBD, please share your agreement or disagreement with the statements below.

	Strongly disagree	Disagree	Neutral	Agree	Strongly agree
Multidisciplinary care is beneficial for pediatric and adolescent patients with IBD.	◯	◯	◯	◯	◯
On average, patients with IBD require more multidisciplinary care than GI patients without IBD.	◯	◯	◯	◯	◯
It is helpful to have multidisciplinary team members accessible during routine clinic visits	◯	◯	◯	◯	◯
Evaluation by multidisciplinary team members at clinic visits should be opt out rather than opt in (e.g. patients are automatically seen by dietician for an assessment even if no specific nutrition related concerns at visit)	◯	◯	◯	◯	◯
Multidisciplinary IBD care should be the standard of care for all patients with IBD	◯	◯	◯	◯	◯
IBD patients and families want multidisciplinary care	◯	◯	◯	◯	◯

Please share how difficult the following items make it to provide multidisciplinary care for children and adolescents with IBD at your center.

	Very difficult	Somewhat difficult	Not at all difficult
Support from divisional leadership	○	○	○
Support from institutional leadership	○	○	○
Clinic space	○	○	○
Provider buy in	○	○	○
Patient buy in	○	○	○
Parent/caregiver buy in	○	○	○
Access to specific multidisciplinary providers	○	○	○
Access to an adequate number of multidisciplinary providers to meet demand	○	○	○
Geographic constraints for patients (i.e. Patients live far from the center, transportation, etc.)	○	○	○
Insurance coverage for multidisciplinary services	○	○	○
Wait times for appointments with multidisciplinary team members	○	○	○
Team dynamics	○	○	○
Other ______	○	○	○

To which of the following is access limited? Check all that apply

- ☐ Nurse practitioner
- ☐ Physician assistant
- ☐ Clinical pharmacist
- ☐ Dietician
- ☐ Nurse
- ☐ Psychologist
- ☐ Social Worker
- ☐ IBD specialized surgeon
- ☐ Case Manager
- ☐ Other ______

Do you ever refer patients with IBD to multidisciplinary team members outside of your center?

- ○ Yes
- ○ No

If yes, which providers? Check all that apply

- ☐ Nurse practitioner
- ☐ Physician assistant
- ☐ Clinical pharmacist
- ☐ Dietician
- ☐ Nurse
- ☐ Psychologist
- ☐ Social Worker
- ☐ IBD specialized surgeon
- ☐ Case Manager
- ☐ Other ______

Do you share multidisciplinary team members with other divisions or departments at your center? For example, a dietitian is shared between GI and endocrinology	◯ Yes<br>◯ No
If yes, which providers? Check all that apply	☐ Nurse practitioner<br>☐ Physician assistant<br>☐ Clinical pharmacist<br>☐ Dietician<br>☐ Nurse<br>☐ Psychologist<br>☐ Social Worker<br>☐ IBD specialized surgeon<br>☐ Case Manager<br>☐ Other ______
Please share any other thoughts or comments you may have regarding the provision of multidisciplinary care for children and adolescents with IBD.	____
